# Bioactive Diterpenoids from *Clerodendrum kiangsiense*

**DOI:** 10.3390/molecules21010086

**Published:** 2016-01-15

**Authors:** Mingfeng Xu, Shengjia Wang, Ouya Jia, Qin Zhu, Lu’e Shi

**Affiliations:** Zhejiang Provincial Key Laboratory for Genetic Improvement and Quality Control of Medicinal Plants, Hangzhou Normal University, Hangzhou 310036, China; zjxmf@163.com (M.X.); shinya33@163.com (S.W.); prospectjoy@sina.com (O.J.); zhuqin@hznu.edu.cn (Q.Z.)

**Keywords:** *Clerodendrum kiangsiense*, abeo-abietane diterpenoid, cytotoxicity

## Abstract

A new abeo-abietane diterpenoid, 12-methoxy-6,11,14,16-tetrahydroxy-17(15→16)-abeo-5,8,11,13-abietatetraen-3,7-dione (**8**), was isolated from the hydroalcoholic extract of the herb of *Clerodendrum kiangsiense* along with seven known diterpenoids (**1**–**7**). Their structures were identified on the basis of spectroscopic analyses including two-dimensional NMR and comparison with literature data. All of these compounds were evaluated for their cytotoxic activities against the growth of human cancer cells lines HL-60, SMMC-7721, A-549 and MCF-7 by the MTT assay. The results showed that cryptojaponol (**4**), fortunin E (**6**) and **8** exhibited significant cytotoxicity against four human cancer cell lines.

## 1. Introduction

Abietane diterpenoids are a class of tricyclic diterpenoids, and have been isolated from plant species from the taxonomic families Verbenaceae, Lamiaceae, Taxaceae [1,2,3,4]. *Clerodendrum* is a genus of about 400 species in the family Verbenaceae, which mainly grows in tropical and warm temperate zones including Africa and Southern Asia. A few species are found in South America, Northern Australia and Eastern Asia [5]. Preparations of the leaves, branches and stems of *Clerodendrum* have been used in folk medicine to treat different diseases such as cancer, catarrhal affections of the lungs, fever, inflammation, skin diseases and asthma [6,7].

**Figure 1:**
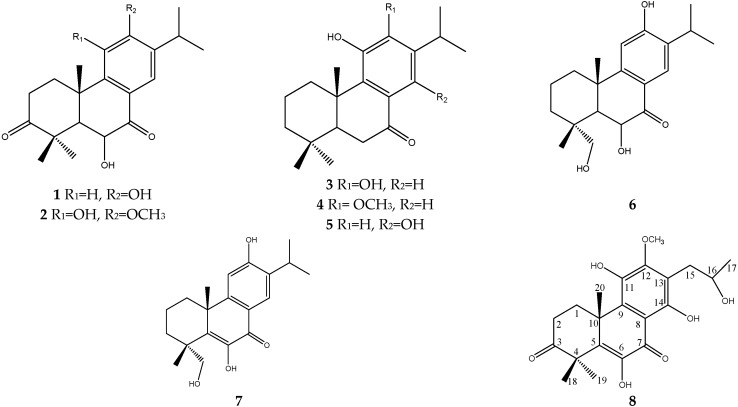
Compounds isolated from *Clerodendrum kiangsiense*.

Phytochemical investigations on *Clerodendrum* species revealed various diterpenoids in the plants, which showed antibacterial and cytotoxic activities [8,9]. However, the phytochemical composition of the stems and roots of *C. kiangsiense* have not been full characterized. As a part of our ongoing effort to discover potential anticancer compounds from Chinese medicinal plants, the stems of *C. kiangsiense*, collected in Jiangxi Province, were investigated systematically. This has led to the isolation and structure elucidation of a new abeo-abietane diterpenoid (**8**) and seven known substances (**1**–**7**) (Figure 1). Compounds **1**–**8** were evaluated for their cytotoxicity against four cancer cell lines.

## 2. Results and Discussion

Compound **8** was isolated as colorless crystals from the EtOAc fraction of the ethanol extract of *C. kiangsiense*, it was assigned the molecular formula C_21_H_26_O_7_ (9 degrees of unsaturation) by HRESIMS (*m*/*z* 389.1597 [M − H]^−^, calcd. for C_21_H_25_O_7_^−^, 389.1599). The absorption bands in the UV spectrum (238, 285, 336, 381 nm) exhibited the presence of a benzene and a ketone. In the IR spectrum, two carbonyl signals were observed at 1660 and 1715 cm^−1^ in addition to the absorption peak at 3405 cm^−1^ for the hydroxyl moiety.

**Figure 2:**
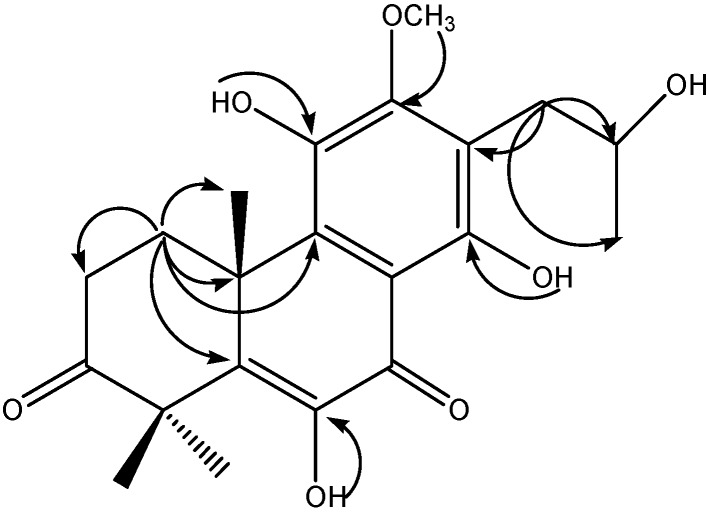
Key HMBC correlations for compound **8**.

The ^1^H- and ^13^C-NMR spectroscopic data (Table 1) indicated the presence of four methyls [δ_H_ 1.28 (3H, d, *J* = 5.5 Hz, H-17), δ_H_ 1.53 (3H, s, H-18), δ_H_ 1.58 (3H, s, H-19) and δ_H_ 1.44 (3H, s, H-20)], a pair of doublet doublets at δ_H_ 1.80, 2.71, 2.73 and 3.32 (m, 1H each) corresponding to two methylene groups, one methine group at δ_H_ 4.18 (1H, m, H-16) together with one methoxyl at δ_H_ group 3.87 (3H, s, OMe-12), two ketone groups at δ_C_ 214.0 (C-3) and δ_C_ 183.6 (C-7), and six aromatic C-atom signals at δ_C_ 109.5, 118.7, 132.9, 139.3, 140.3 and 142.4. These data, together with other spectroscopic characteristics, suggested that **8** was a diterpenoid [10]. ^1^H-^1^H COSY correlations were observed from: H-1 (δ_H_ 1.80 and δ_H_ 3.32) to H-2 (δ_H_ 2.71 and δ_H_ 2.73); and H-17 (δ_H_ 1.28) through H-16 (δ_H_ 4.18) to H-15 (δ_H_ 2.83 and δ_H_ 2.91), in combination with HMBC correlations (Figure 2) between: H-20 (δ_H_ 1.44) to C-1 (δ_C_ 26.9), C-5 (δ_C_ 140.3), C-9 (δ_C_ 139.3), and C-10 (δ_C_ 40.6); H-15 (δ_H_ 2.83 and δ_H_ 2.91) to C-12 (δ_C_ 152.6), C-13 (δ_C_ 118.7), C-14 (δ_C_ 155.2), C-16 (δ_C_ 67.8) and C-17 (δ_C_ 23.8), which suggested that the oxygenated substituent was placed at the C-16 position (–CH_2_CH(OH)CH_3_), and the side chain of **8** is not an isopropyl but rather a 2-hudroxy-*n*-propyl group(CH_3_-17 shifted to C-16 from C-15); OH-6 (δ_H_ 6.86) to C-6 (δ_C_ 142.4) and C-7 (δ_C_ 183.6); OH-11 (δ_H_ 5.97) to C-11 (δ_C_ 132.9); OH-14 (δ_H_ 12.56) to C-8 (δ_C_ 109.5), C-13 (δ_C_ 118.7) and C-14 (δ_C_ 155.2); The ^1^H,^13^C long-range correlations between H-21 (δ_H_ 3.87) to C-12 (δ_C_ 152.6), therefore, compound **8** possesses an abeo-abietane diterpenoid framework with an OCH_3_ group on C-12. Thus, the structure of **8** was elucidated as 12-methoxy-6,11,14,16-tetrahydroxy-17(15→16)-abeo-5,8,11,13- abietatetraen-3,7-dione. (The NMR spectra of compound 8 are listed in the Supplementary Materials).

The seven known compounds were identified as mandarone A (**1**) [11], taxusabietane A (**2**) [12], 12-O-demethylcrypto-japonol (**3**) [13], cryptojaponol (**4**) [14], 11,14-dihydroxy-8,11,13-abietatrien-7-one (**5**) [15], fortunin E (**6**) [16], fortunin F (**7**) [16], by comparison of their spectra with those reported in the literature.

**Table 1:** NMR spectroscopic data for compound **8** in CDCl_3_.

NO	δ_H_ (*J* in Hz)	δ_C_	HMBC
1	1.80, m, α 3.32, m, β	26.9, CH_2_	C-2, C-3, C-10, C-20
2	2.71, m, α 2.73, m, β	33.2, CH_2_	C-1, C-3, C-4, C-10
3		214.0, qC	
4		48.8, qC	
5		140.3, qC	
6		142.4, qC	
7		183.6, qC	
8		109.5, qC	
9		139.3, qC	
10		40.6, qC	
11		132.9, qC	
12		152.6, qC	
13		118.7, qC	
14		155.2, qC	
15	2.91, dd (13.8, 4.0) ^a^ 2.83, dd (13.7, 8.3) ^a^	32.9, CH_2_	C-12, C-13, C-14, C-16, C-17
16	4.18, m	67.8, CH	C-15, C-17
17	1.28, d (6.2)	23.8, CH_3_	C-15, C-16
18	1.58, s	21.0, CH_3_	C-3, C-4, C-5, C-19
19	1.53, s	24.3, CH_3_	C-3, C-4, C-5, C-18
20	1.44, s	20.0, CH_3_	C-1, C-5, C-9, C-10
21-OCH_3_	3.87, s	61.7,OCH_3_	C-12

^a^ Assignments may be reversed.

To investigate their cytotoxic activities, these compounds were evaluated using an MTT cytotoxicity assay against human myeloid leukemia (HL-60), hepatocellular carcinoma (SMMC-7721), lung cancer (A-549) and breast cancer (MCF-7) cell lines. The IC_50_ values of all eight compounds against the indicated cancer cells are summarized in Table 2. Compound **6** and **8** exhibited the strongest cytotoxicity to all cells, as its range of IC_50_ values was 1.8–5.0 μM. Additionally, A-549 was the most sensitive cell line to these types of compounds among all tested cancer cells because the IC_50_ values of all compounds against A-549 cells were close to 10 μM. Furthermore, the cytotoxicities of all the isolated compounds were comparable to the chemotherapeutic drug cisplatin [17], which suggests that these compounds might have promising potential to be anticancer agents.

**Table 2:** Cytotoxic activities of compounds **1**–**8** isolated from *C. kiangsiense* (IC_50_ in μM).

Compounds	HL-60	SMMC-7721	A-549	MCF-7
**1**	12.5 ± 1.2	13.6 ± 0.8	7.4 ± 1.1	27.7 ± 2.2
**2**	18.6 ± 1.8	15.9 ±1.6	10.2 ± 1.2	15.4 ± 2.7
**3**	23.5 ± 2.0	33.0 ± 2.4	10.4 ± 1.3	19.2 ± 1.8
**4**	9.9 ± 0.9	6.7 ± 1.0	8.7 ± 0.8	10.0 ± 0.8
**5**	15.5 ± 1.9	15.7 ± 1.6	11.8 ± 2.4	22.4 ± 2.9
**6**	4.8 ± 0.5	3.8 ± 0.9	2.7 ± 0.7	5.0 ± 1.0
**7**	15.7 ± 1.8	5.8 ± 0.8	7.9 ± 0.7	19.2 ± 1.4
**8**	1.8 ± 0.3	4.9 ± 0.7	2.5 ± 0.7	3.1 ± 0.5
Cisplatin	4.2 ± 0.5	5.9 ± 0.9	9.8 ± 1.1	11.3 ± 1.0

Results are expressed as means of IC_50_ values (the concentration that reduced cell growth by 50%) in μM, and data were obtained from triplicate experiments.

## 3. Materials and Methods

### 3.1. General Experimental Procedures

Melting points were measured using a XT-4 micro melting point apparatus (Beijing, China). Optical rotations were determined at 25 °C on a JASCO (Tokyo, Japan) P2000 polarimeter. UV data were measured using a Shimadzu UV-2550 spectrophotometer (Shimadzu, Kyoto, Japan). IR spectra were recorded on a Nicolet 380 FT-IR spectrophotometer (Thermo Fisher Scientific, Waltham, MA, USA). NMR spectra were recorded on a Bruker Avance III 500 spectrometer (Bruker, Bremen, Germany), using TMS as internal standard, Chemical shifts are reported as δ values and the coupling constants (*J*) are in Hz. HRESIMS data were obtained on a Agilent 6210 TOF-MS mass spectrometer; HPLC (Amersham Biosciences, GE Healthcare Life Science, Santa Clara, CA, USA), Waters 1525 semi-preparative HPLC system (Waters Co. Ltd., Milford, MA, USA) coupled with a Waters 2996 photodiode array detector. A Kromasil C18 preparative HPLC column (250 × 10 mm, 5 μm) was used; Column chromatography was carried out on silica gel (Qing Dao Hai Yang Chemical Group Co., Qingdao, China; 200–300 mesh) and Sephadex LH-20 (Amersham Biosciences). TLC analyses were carried out on silica gel 60 F_254_ (Merck, Darmstadt, Germany) and RP-18 F_254s_ (Merck) plates. Compounds were detected by UV and 30% H_2_SO_4_ spraying reagent followed by heating at 105 °C for 1–2 min.

### 3.2. Plant Material

The stems of *C. kiangsiense* were collected on the Wugong Mountain of Pingxiang City, Jiangxi Province, China, in September 2010, and identified by Chunhui Dai in Zhejiang Academy of Traditional Chinese Medicine. A voucher specimen (No. 201007) has been deposited in the Key Laboratory for Genetic Improvement and Quality Control of Medical Plants of Zhejiang Province.

### 3.3. Extraction and Isolation

The air-dried powder of the stems (7.6 kg) of *C. kiangsiense* was extracted by 90% ethanol (30 L × 3) at 65 °C. The solvents were combined and evaporated to dryness under vacuum at 50 °C to afford a gummy residue (110 g). This residue was suspended in H_2_O (1000 mL) and partitioned with petroleum ether (1000 mL × 3, 19 g), ethyl acetate (EtOAc) (1000 mL × 3, 32 g), and *n*-BuOH (1000 mL × 3, 9 g), successively. The EtOAc fraction (19 g) was subjected to silica-gel column chromatography (CC) with a step gradient elution of petroleum ether–EtOAc (20:1 to 1:4, *v*/*v*) to afford 8 fractions (Fr.1–Fr.8). Fr.2 was repeatedly separated by silica gel column chromatography eluted with a petroleum ether–EtOAc gradient (20:1 to 4:1, *v*/*v*) to give compound **1** (13 mg), compound **3** (7 mg) and a fraction that was a mixture of three compounds. Further chromatographic purification on Sephadex LH-20 CC with a methanol afforded compound **2** (9 mg), compound **8** (9 mg) and compound **4** (10 mg). The separation of Fr.3 on silica gel eluted with EtOAc:petroleum ether (10:1 to 2:1, *v*/*v*) afforded a mixture (40 mg) of compounds **5** (7 mg), **6** (7 mg) and **7** (16 mg), which was separated by prep-HPLC using an acetonitrile–water mixture (30:70 *v*/*v*).

Compound **8**: colorless crystals; m.p. 284–285 °C; ${\left[\alpha \right]}_{D}^{25}$ +15.7° (*c* 0.1, MeOH); UV λ_max_: 238, 285, 336, 381 nm; IR (KBr) ν_max_: 3405, 1715, 1660, 1605, 1581, 1500, 1457, 1382, 1331 cm^−1^; ^1^H- and ^13^C-NMR (CDCl_3_) spectroscopic data, see Table 1; HRESIMS (negative ion mode) *m*/*z* 389.1597 [M − H]^−^ (calcd. for C_21_H_25_O_7_^−^, 389.1599).

### 3.4. Anti-Proliferative Activity

The percentage of growth inhibition was determined using a MTT colorimetric technique to measure four viable cells (HL-60 human myeloid leukemia, SMMC-7721 hepatocellular carcinoma, A-549 lung cancer and MCF-7 breast cancer) with minor modification [18,19]. A total of 5000–10,000 exponential phase cells per well were seeded onto a 96-well plate for 24 h, Each tumor cell line was exposed to a test compound at concentrations of 0.0624, 0.32, 1.6, 8, and 40 μM in DMSO in triplicate for 72 h, with cisplatin as the positive control. Briefly, 100 μL of a MTT working solution (1 mg/mL) was added into each well and incubated at 37 °C for 4 h and then the medium was removed. The converted dye formazan was solubilized with 150 μL acidic isopropanol (0.04 M HCl in absolute isopropanol) and each concentration was tested in triplicate. The absorbance was then measured at a wavelength of 570 nm using a microplate reader (Multiskan Spectrum, Thermo Electron Corporation, Vantaa, Finland). The dose resulting in 50% inhibition of cell growth (IC_50_) was calculated by the Reed and Muench method.

## 4. Conclusions

In the present study, a new abeo-abietane diterpenoid, 12-methoxy-6,11,14,16-tetrahydroxy-17(15→16)-abeo-5,8,11,13-abietatetraen-3,7-dione (**8**) was isolated from *C. kiangsiense* by chromatographic separation of a 90% EtOH extract of its stems. In addition, 7 known compounds (**1**–**7**) were also obtained. The structures of compounds **1**–**8** were determined by spectroscopical data interpretation. The isolated compounds were subsequently evaluated for cytotoxic activities against HL-60 human myeloid leukemia, SMMC-7721 hepatocellular carcinoma, A-549 lung cancer and MCF-7 breast cancer cells, respectively. Compounds **4**, **6** and 8 exhibited the strongest cytotoxicity to all cells. Additionally, A-549 was the most sensitive cell line to these types of compounds among all tested cancer cells. Furthermore, the cytotoxicities of the isolated compounds were comparable to the chemotherapeutic drug cisplatin, which suggests that *C. kiangsiense* and its constituents could be useful sources of candidates for the development of anticancer medicines.

## Supplementary Materials

Supplementary materials can be accessed at: http://www.mdpi.com/1420-3049/21/1/86/s1.
