# Supplementary material for: Bioactive Diterpenoids from Clerodendrum kiangsiense
Source: Molecules. 2016 Jan 15;21(1):86. doi: 10.3390/molecules21010086 (PMC6273191; doi:10.3390/molecules21010086)
Supplement: Supplementary file 1 [file molecules-21-00086-s001.pdf]

Supplementary Materials: Bioactive Diterpenoids from *Clerodendrum kiangsiense*

Mingfeng Xu, Shengjia Wang, Ouya Jia, Qin Zhu and Lu'e Shi

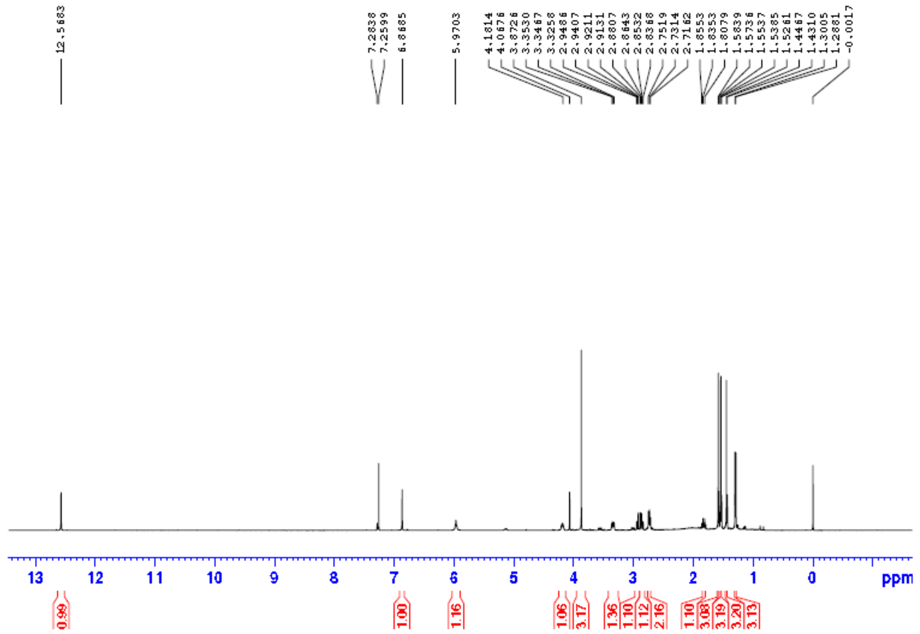

Figure S1. ^{1}H-NMR of Compound **8**.

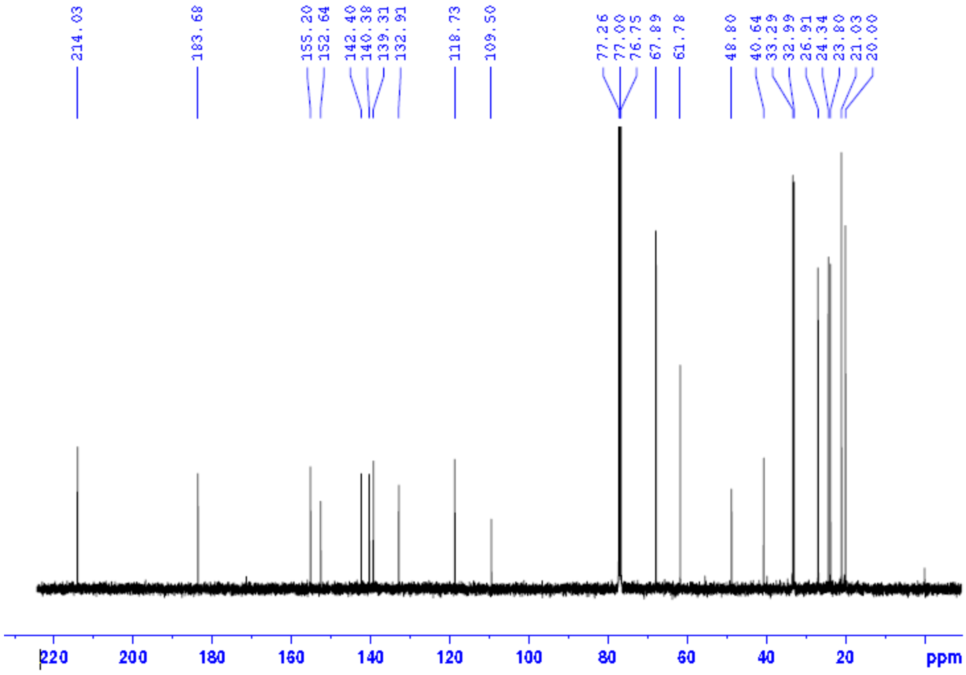

Figure S2. ^{13}C-NMR of Compound **8**.

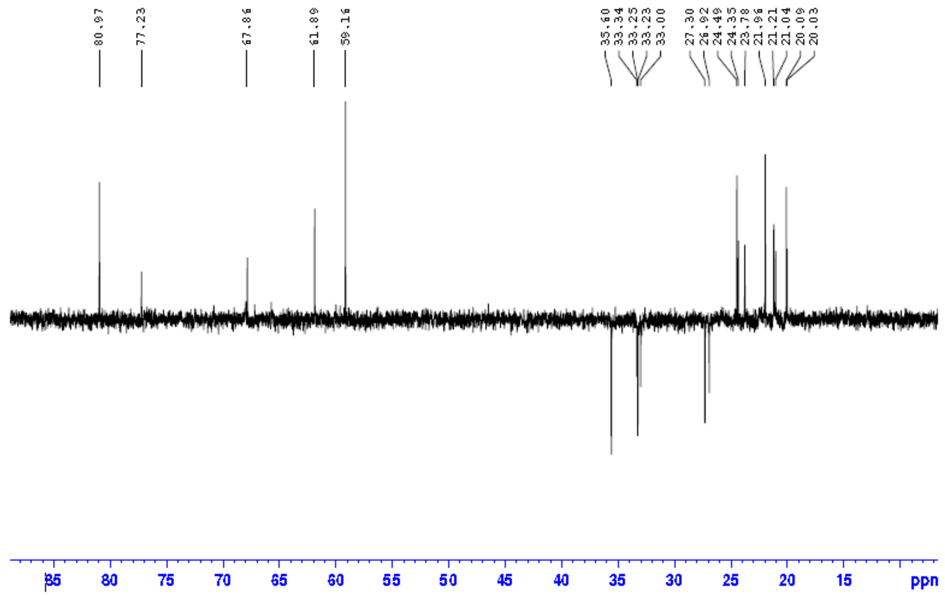

Figure S3. DEPT 135 of Compound **8**.

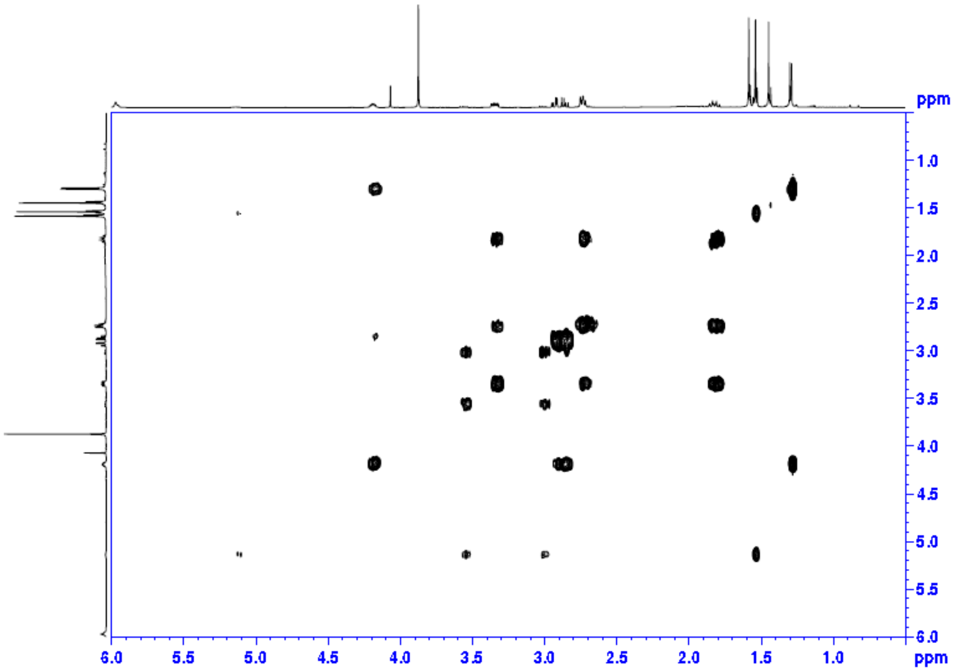

Figure S4. ^{1}H-^{1}H COSY of Compound **8**.

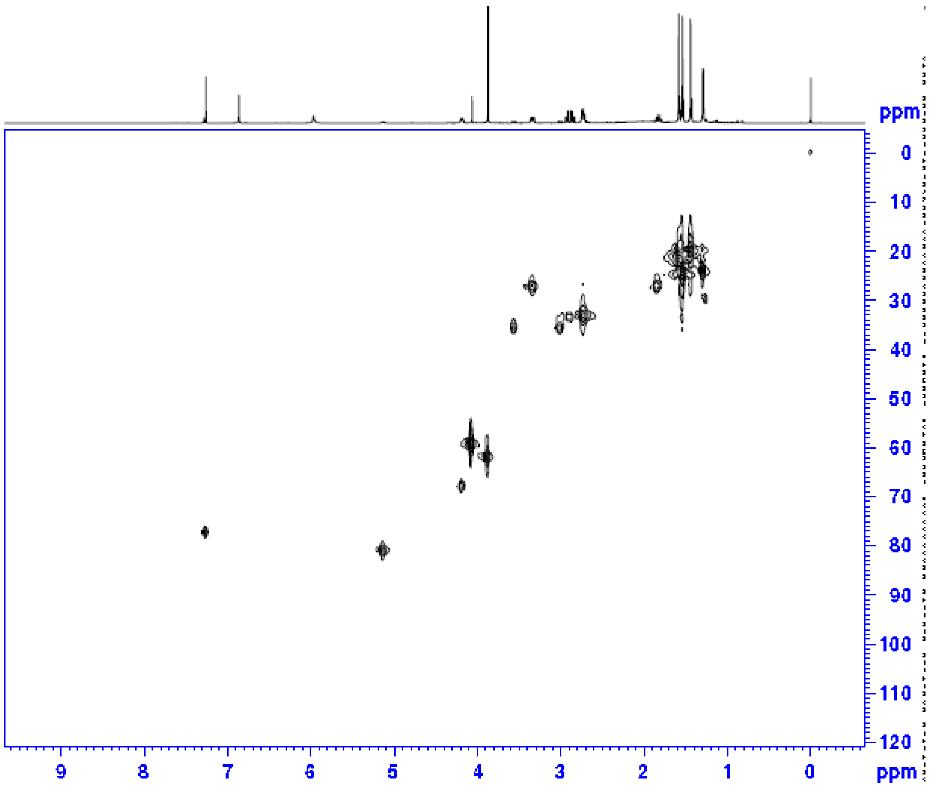

Figure S5. HSQC of Compound **8**.

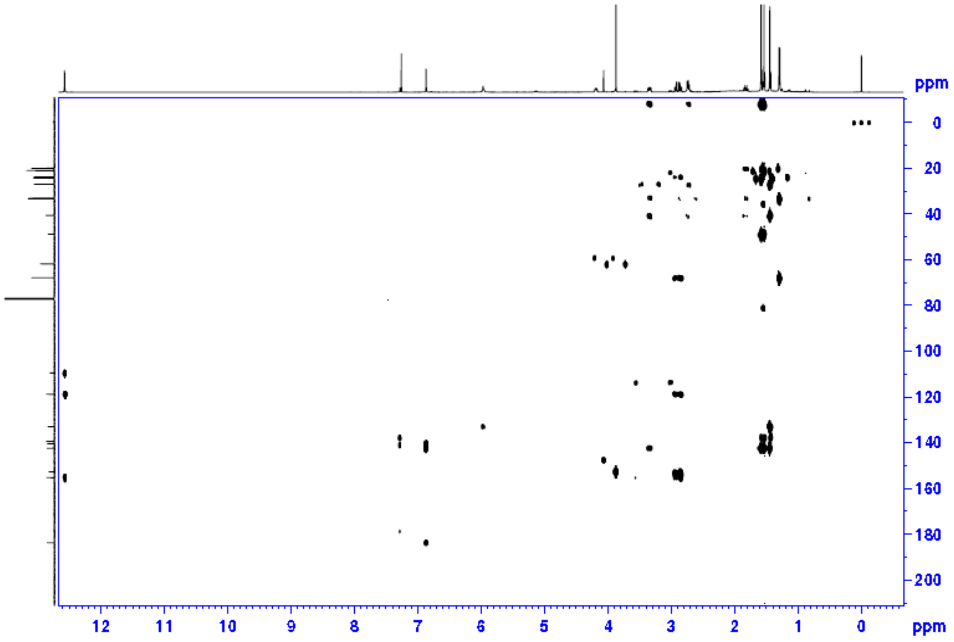

Figure S6. HMBC of Compound **8**.
